# Editorial: Exploring macrophage roles in cancer progression and therapeutic targeting

**DOI:** 10.3389/fimmu.2025.1612588

**Published:** 2025-05-08

**Authors:** George S. Karagiannis, Yu Kato, David Entenberg

**Affiliations:** ^1^ Integrated Imaging Program for Cancer Research, Albert Einstein College of Medicine, Montefiore Medical Center, Bronx, NY, United States; ^2^ Cancer Dormancy Institute, Albert Einstein College of Medicine, Montefiore Medical Center, Bronx, NY, United States; ^3^ Department of Microbiology and Immunology, Albert Einstein College of Medicine, Montefiore Medical Center, Bronx, NY, United States; ^4^ Gruss-Lipper Biophotonics Center, Albert Einstein College of Medicine, Montefiore Medical Center, Bronx, NY, United States; ^5^ Tumor Microenvironment and Metastasis Program, Albert Einstein College of Medicine, Montefiore Medical Center, Bronx, NY, United States; ^6^ Tsukuba Research Laboratories, Eisai Co., Ltd., Tsukuba, Ibaraki, Japan; ^7^ Department of Pathology, Albert Einstein College of Medicine, Montefiore Medical Center, Bronx, NY, United States

**Keywords:** macrophages, cancer, metastasis, immunotherapy, cancer progression

## Introduction

Macrophages are versatile cells which play pivotal roles in development, tissue repair, and immune response. Lying at the intersection between the innate and adaptive immune systems, these cells serve as sentinels, surveilling tissues for damage, infection, or oncogenic transformation (1, 2). Within cancer microenvironments, these versatile cells transform into tumor-associated macrophages (TAMs) or metastasis-associated macrophages (MAMs), and exhibit a dual nature capable of both promoting antitumor immunity and facilitating cancer progression (3).

Within the primary tumor microenvironment, TAMs are known to facilitate tumor malignancy through various mechanisms: promoting angiogenesis, suppressing antitumor immunity, and enhancing tumor cell invasion and metastatic dissemination. Meanwhile, at secondary sites, MAMs also play a crucial role in the metastatic process, aiding in tumor cell extravasation, survival, and growth. Understanding the complexity of macrophage biology, and how to modulate this biology to inhibit tumor growth and metastasis, is a key focus of current research efforts.

Despite recent advances in cancer immunotherapy (e.g. immune checkpoint inhibition), refractory cancers remain a significant challenge. This persistence highlights the need for novel therapeutic approaches. Targeting macrophages and their associated molecules has emerged as a potential strategy for both anticancer and antimetastatic therapies. However, a comprehensive understanding of macrophage biology in cancer initiation and metastasis is still lacking, necessitating further investigation into their roles and therapeutic potential.

This Research Topic highlights key advances in our understanding of macrophage biology in cancer, focusing on their heterogeneity, molecular mechanisms, and therapeutic potential. Several contributions to this Research Topic showcase recent studies using single-cell and spatial technologies that reveal diverse macrophage subpopulations with context-dependent roles in tumor progression. Additionally, papers included in this Research Topic investigate signaling pathways, metabolism, and intercellular communication, shedding light on how macrophages influence cancer dynamics. Finally, several papers propose innovative strategies to reprogram or target tumor-associated macrophages, offering promising avenues for cancer therapy. Together, this Research Topic will be valuable to researchers and clinicians seeking to develop macrophage-targeted treatments and better understand the immune landscape of tumors.

## Contributions to the Research Topic

### Heterogeneity and plasticity of macrophages within the tumor microenvironment

The tumor microenvironment is a complex ecosystem where macrophages exhibit remarkable diversity and adaptability. Several contributions include a major focus on the critical role of macrophage heterogeneity and plasticity in cancer progression and immunotherapy, underscoring the need for a nuanced understanding of their function.

The review by Stavrou et al. summarizes recent findings on distinct TAM subsets in the tumor microenvironment and their involvement in breast cancer progression, emphasizing the constant interplay between TAMs and breast cancer cells as a major contributor to disease progression. This interaction involves the polarization of macrophages toward a tumor-promoting phenotype, induction of epithelial-to-mesenchymal transition in cancer cells, and enhancement of cancer stem cell properties. The authors discuss the clinical relevance of these findings, focusing on how a better understanding of TAM involvement in breast cancer metastasis could lead to more effective treatment options. They suggest that a thorough characterization of individual patients’ TAM signatures could facilitate the design of personalized treatment strategies and improve the prediction of treatment responses.

The paper by Zhou et al. provides an overview of the role of macrophages in cancer immunotherapy, highlighting recent advances in understanding their complex functions within the tumor microenvironment. Macrophages are a major component of the immune infiltrate in many solid tumors and can exhibit both pro- and anti-tumor activities depending on their phenotype and the signals they receive. The review discusses how TAMs often adopt an immunosuppressive, pro-tumor phenotype that promotes cancer progression and metastasis. However, emerging research has revealed strategies to reprogram these TAMs or harness their anti-tumor potential for cancer therapy. The authors describe several approaches being explored to target macrophages in cancer immunotherapy. These include blocking macrophage recruitment to tumors, depleting TAMs, repolarizing TAMs from a pro-tumor M2-like phenotype to an anti-tumor M1-like phenotype, and enhancing macrophage phagocytosis of cancer cells. The paper also discusses combining macrophage-targeted therapies with other immunotherapies like immune checkpoint inhibitors. Additionally, it highlights new technologies like single-cell RNA sequencing that are providing deeper insights into macrophage heterogeneity and function in the tumor microenvironment. Overall, the review emphasizes that macrophages represent a promising but complex target for improving cancer immunotherapy, with ongoing research aimed at better understanding and manipulating their diverse roles in tumors.

### Elucidation of molecular mechanisms by which macrophages contribute to cancer progression

Beyond understanding the broad role of macrophage heterogeneity, many contributions have focused on elucidating the specific molecular mechanisms by which macrophages contribute to cancer progression. This includes investigating the intricate signaling pathways, interactions, and molecules that govern macrophage behavior within the tumor microenvironment.

In the review article by Baig et al., the authors explore the role of adaptor proteins in regulating inflammation in macrophages. Adaptor proteins are non-catalytic proteins that act as molecular bridges between cell surface receptors and intracellular effector molecules, mediating protein-protein interactions and modulating immune cell signaling. These proteins play critical roles in organizing signaling complexes, regulating protein localization, and modulating the intensity/duration of cellular responses. Some adaptor proteins can function to activate signaling pathways, while others inhibit them. This dichotomy offers an opportunity to affect and alter macrophage function.

As such, this article comprehensively reviews 20 adaptor molecules that actively dampen inflammatory signaling pathways in macrophages. The authors discuss how these adaptor proteins regulate signal transduction processes, driving macrophages from pro-inflammatory M1-like states to anti-inflammatory M2-like phenotypes. By mapping the specific functions and structural domains of these molecules, the review illuminates their complex interplay in immune regulation. This work focuses on our current understanding of adaptor dynamics but also paves the way for therapeutic strategies targeting chronic inflammatory conditions, offering new investigative avenues for clinical applications in diseases marked by persistent inflammation.

Another way that TAMs are able to impact tumor cells is by imparting in them aggressive phenotypes (such as increased motility, invasiveness, and epithelial-mesenchymal transition (EMT)). The article by DeLuca et al. discusses the various molecular mechanisms by which TAMs facilitate tumor cell migration and invasion. These include the secretion of proteolytic enzymes that degrade the extracellular matrix, the production of growth factors and cytokines that stimulate tumor cell motility, and direct interactions with tumor cells that guide their movement. Recent evidence indicates that these factors and interactions may be amplified by traditional anti-tumoral therapies, potentially leading to the emergence of prometastatic phenotypes in tumor cells. The authors continue on to describe how host factors such as diet, race, and obesity, can influence macrophages and their ability to support or counter tumor development.

The review by Murrey et al. views macrophages with a different lens, focusing on the critical role of macrophage motility and migration in various physiological and pathological processes. Macrophages, as tissue-resident immune cells, are highly motile and continuously patrol their environment to maintain tissue homeostasis, respond to injury or infection, and participate in development. The authors discuss the diverse mechanisms that regulate macrophage migration, including chemotactic signals, adhesion molecules, and cytoskeletal dynamics. They highlight how these mechanisms are tightly controlled to ensure proper macrophage recruitment and function in different contexts.

This review also explores the role of macrophage migration in tumor invasion and metastasis. TAMs can promote cancer cell migration and invasion by secreting growth factors, matrix-degrading enzymes, and chemokines. The authors discuss how TAMs are recruited to the tumor microenvironment and how their migratory behavior contributes to tumor progression. They also highlight potential therapeutic strategies for targeting macrophage migration to inhibit tumor invasion and metastasis.

The study by Xie et al. investigates the characteristics and potential antitumor functions of immortalized bone marrow-derived macrophages (iBMDMs) compared to primary bone marrow-derived macrophages (BMDMs) and the RAW264.7 cell line. The researchers found that iBMDMs exhibit similar macrophage biomarkers and polarization responses to BMDMs and RAW264.7 cells, with the ability to polarize into M1 and M2 phenotypes upon appropriate stimulation. iBMDMs demonstrated rapid proliferation and long-term survival both *in vitro* and *in vivo*, while maintaining biosafety in mouse tissues. Importantly, iBMDMs showed strong phagocytic capacity against tumor cells, especially after M1 polarization.

The study also revealed that iBMDMs have potent antitumor effects through various mechanisms. The supernatant from M1-polarized iBMDMs significantly inhibited tumor cell proliferation and promoted apoptosis of tumor cells. Additionally, iBMDMs, particularly M1-polarized ones, demonstrated a remarkable ability to inhibit tumor cell migration by suppressing EMT. *In vivo* experiments showed that M1-polarized iBMDMs could maintain their anti-tumor phenotypes and influence recruited macrophages in recipient mice, leading to improved tumor immune microenvironments and repressed tumor growth. These findings suggest that iBMDMs can serve as a valuable tool for studying macrophage functions and mechanisms, as well as a potential source for macrophage-based immunotherapy in cancer treatment.

The study by Yang et al. investigates the influence of sex disparities on macrophage proliferation and accumulation in hepatocellular carcinoma (HCC). The researchers found higher levels of macrophage density and proliferation in tumor tissues from male HCC patients compared to females. They discovered that the expression of G protein-coupled estrogen receptor 1 (GPER1), a non-classical estrogen receptor, was significantly decreased in proliferating macrophages and inversely correlated with macrophage proliferation in HCC tumors. Activation of GPER1 signaling with a selective agonists, G1, suppressed macrophage proliferation by downregulating the MEK/ERK pathway.

Furthermore, G-1 treatment reduced PD-L1 expression on macrophages and delayed tumor growth in mice. The study also found that patients with a higher percentage of GPER1+ macrophages exhibited longer overall survival and recurrence-free survival compared to those with lower levels. These findings reveal a novel role of GPER1 signaling in regulating macrophage proliferation and function in HCC tumors. The research suggests that understanding sex-related disparities in patients may offer potential strategies for designing more effective therapies for HCC.

### Development of novel therapeutic strategies targeting macrophages in cancer treatment

Recognizing the significant impact of macrophages on cancer progression, researchers are actively exploring novel therapeutic strategies to target these cells within the tumor microenvironment. The following studies highlight diverse approaches aimed at manipulating macrophage number and function to improve cancer treatment outcomes.

The review by Cao et al. delves into the major signaling pathways through which TAMs can either promote or suppress tumor progression, and the multifaceted strategies of targeting them for cancer treatment. These immunotherapeutic approaches attempt to alter TAMs from a pro-tumorigenic (M2-like) to an anti-tumorigenic (M1-like) phenotype by blocking M2 macrophage recruitment, depleting them, or modulating their functions to enhance the efficacy of cancer therapies. The authors discuss various mechanisms by which TAM-targeted immunotherapies attempt to exert their effects, including altering cytokine production, enhancing antigen presentation, and promoting cytotoxic T cell infiltration. They also address the challenges associated with TAM-targeted approaches, such as the heterogeneity of TAMs, their plasticity, and the potential for off-target effects. Finally, the review emphasizes the importance of mechanistic studies to better understand the complex interactions between TAMs and cancer cells, as well as the rational design of more effective and selective TAM-targeted immunotherapies.

The paper Lin et al. continues the discussion of how macrophages can play a dual role in cancer progression, but with a particular focus on head and neck squamous cell carcinoma (HNSCC). The authors discuss several modes of tumor cell-macrophage interaction, including phagocytosis and the secretion of cytokines and exosomes. They discuss the potential of macrophages as both diagnostic and therapeutic targets in HNSCC and the various strategies for targeting TAMs in this carcinoma. These strategies include reprogramming macrophages towards an anti-tumor phenotype, inhibiting macrophage recruitment, and combining macrophage-targeted therapies with conventional treatments. The authors also highlight the use of macrophage-related markers for prognostic and diagnostic purposes in HNSCC. Overall, the paper underscores the importance of understanding macrophage biology in the context of HNSCC to develop more effective treatment strategies and improve patient outcomes.

The primary research article by Schultze-Rhonhof et al. investigates the effects of Plasma-activated liquids (PALs) on human tissue-resident peritoneal macrophages. PALs are an emerging technology with promising applications in medicine and biomedical research. PALs are created by exposing liquids like water or growth media to atmospheric plasma discharges. This process generates long-lived reactive species such as hydrogen peroxide, nitrites, and nitrates, as well as short-lived species like hydroxyl radicals and peroxynitrite. PALs have demonstrated significant potential in wound healing, cancer treatment, and antimicrobial applications due to their ability to induce oxidative stress in target cells while minimizing damage to healthy tissues.

The researchers isolated primary human macrophages from the peritoneum and exposed them to PALs. Using various methods including flow cytometry, Raman microspectroscopy, and DigiWest protein analysis, the study found that macrophages demonstrated a pronounced resistance to PALs, characterized by an upregulation of proliferation and anti-oxidative pathways to counter PAL-derived oxidative stress-induced cell death.

The findings revealed that PAL treatment led to changes in the macrophages’ lipid composition and a moderate increase in pro-inflammatory cytokine release. However, the macrophages maintained high viability and showed minimal levels of apoptosis and necrosis. The researchers suggest that these cellular effects of PAL on human tissue-resident peritoneal macrophages could potentially lead to immunomodulatory effects within the human peritoneal cavity. This study contributes to understanding the interaction between PALs and macrophages, highlighting promising prospects for PALs in the adjuvant treatment of peritoneal cancer.

The paper by Wei et al. reviews the potential of natural plant-derived polysaccharides as modulators of macrophage polarization for cancer immunotherapy. Plant polysaccharides have shown promise in regulating macrophage polarization, particularly in promoting the M1 phenotype and inhibiting the M2 phenotype. The review covers the classification, sources, and mechanisms of action of these polysaccharides, including their effects on cytokine production, NO and ROS generation, and activation of signaling pathways such as TLR4, MAPK, and NF-κB.

The paper also explores the clinical translation and application of plant polysaccharides, focusing on compounds like Astragalus polysaccharide and Belapectin. These substances have shown potential in enhancing the efficacy of chemotherapy and immunotherapy in various cancer types. However, the authors note that challenges remain in the clinical translation of plant polysaccharides, including the need for more defined extracts and further research into optimal dosing and potential side effects. The review concludes by highlighting the promise of plant polysaccharides as immunomodulators in cancer therapy while acknowledging the need for further investigation into their specific molecular mechanisms and direct targets.

Finally, the paper by Zhang et al. reviews the role of TAMs in HCC. The authors highlight that TAMs are a major component of the tumor microenvironment in HCC and play a crucial role in tumor progression, metastasis, and therapeutic resistance. TAMs in HCC are primarily derived from circulating monocytes and are polarized towards an M2-like phenotype that promotes tumor growth. The review summarizes how TAMs contribute to HCC development through various mechanisms, including promoting angiogenesis, suppressing anti-tumor immunity, enhancing tumor cell proliferation and invasion, and facilitating metastasis.

The paper also discusses potential therapeutic strategies targeting TAMs in HCC. These include inhibiting TAM recruitment, reprogramming TAMs from a pro-tumor to an anti-tumor phenotype, and depleting TAMs from the tumor microenvironment. The authors review several promising approaches being investigated, such as CSF1R inhibitors, CCR2 antagonists, and CD47 blockade. They emphasize that combining TAM-targeted therapies with other treatments like immune checkpoint inhibitors or anti-angiogenic drugs may be particularly effective for treating HCC. Overall, the review underscores the importance of TAMs as both key drivers of HCC progression and promising therapeutic targets.

## Conclusion

The contributions to this Research Topic collectively advance our understanding of macrophage biology in cancer initiation and metastasis. They align closely with the theme of exploring macrophages as potential therapeutic targets and provide valuable insights into the complex roles these cells play in cancer progression.

These studies highlight the heterogeneity and plasticity of macrophages in the tumor microenvironment, the importance of macrophage-derived factors in promoting metastasis, and the potential of targeting macrophages to overcome therapy resistance. The development of new tools, such as iBMDMs hold the potential to facilitate further research in this field.

As we continue to unravel the intricacies of macrophage biology in cancer, studies of novel therapeutics will pave the way towards improved outcomes for patients with refractory cancers. Future research building on these contributions will be crucial in translating our growing understanding of macrophages in cancer into effective clinical interventions.
